# Supplementary material for: Odor-evoked increases in olfactory bulb mitral cell spiking variability
Source: iScience. 2021 Aug 4;24(9):102946. doi: 10.1016/j.isci.2021.102946 (PMC8397902; doi:10.1016/j.isci.2021.102946)
Supplement: Document S1. Figures S1–S4 [file mmc1.pdf]

iScience, Volume *24*

Supplemental information

Odor-evoked increases in olfactory bulb mitral cell spiking variability

Cheng Ly, Andrea K. Barreiro, Shree Hari Gautam, and Woodrow L. Shew

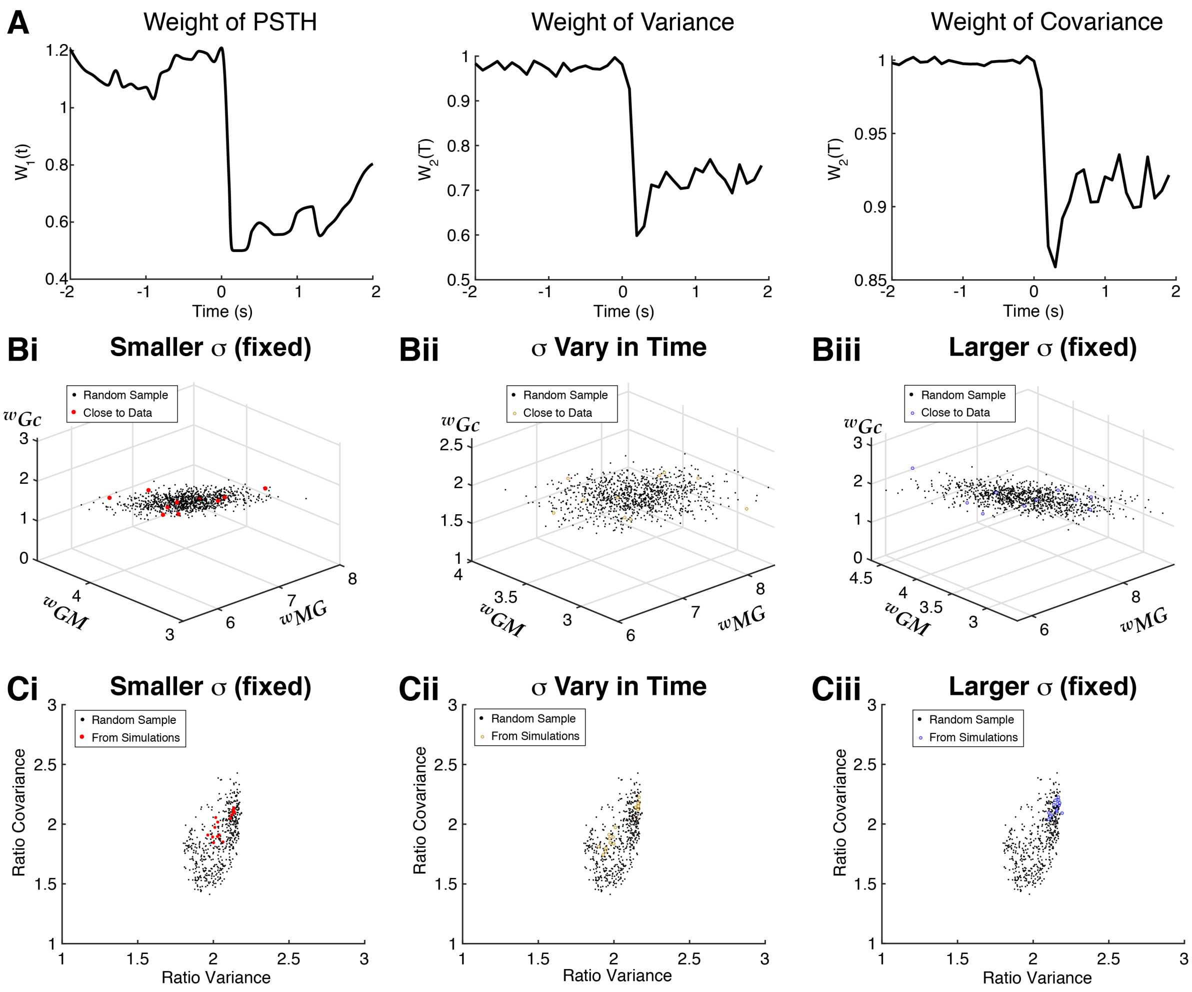

Figure S1: **Details for error calculations, Related to Figures 2, 3, 4. A)** Time-varying weights to calculate error for each of the spike statistics for anesthetized data only (weights are all 1 for awake data). We choose a monotonically decreasing function of the standard deviation of the data (across population / pairs). The weight of the PSTH is $W_1(t) = 1 + 0.5 \tanh\left((std(t) - 10)/5\right)$, the spike count variance is $W_2(T) = 1 + 0.5 \tanh\left((std(T) - 1)/5\right)$, covariance is: $W_3(T) = 1 + 0.5 \tanh\left((std(T) - 0.2)/5\right)$. **B)** Black dots are random samples of coupling strength parameters preserving relationships of parameters that best match time-varying statistics of data for the 3 input noise regimes (10 best parameters in Fig. 3**D** are in red, tan, and blue), used for results in Fig. 4. **C)** Scaling factors from simulations (red, tan and blue circles, again from 10 best parameters) relating instantaneous spike count variance and covariance to spike statistics in 100 ms time window. Horizontal axis shows ratio of spike count variance in instantaneous window (dt) to 100 ms time window, vertical shows same ratio for covariance. Each parameter set has 2 ratios: time-averaged ratios in spontaneous state, and time-averaged ratios in the evoked state. We use a single large random sample of ratios (black dots are same for all 3 panels) preserving the dependencies between the variance and covariance ratios; used for results in Fig. 4. Code to generate all random samples on GitHub.

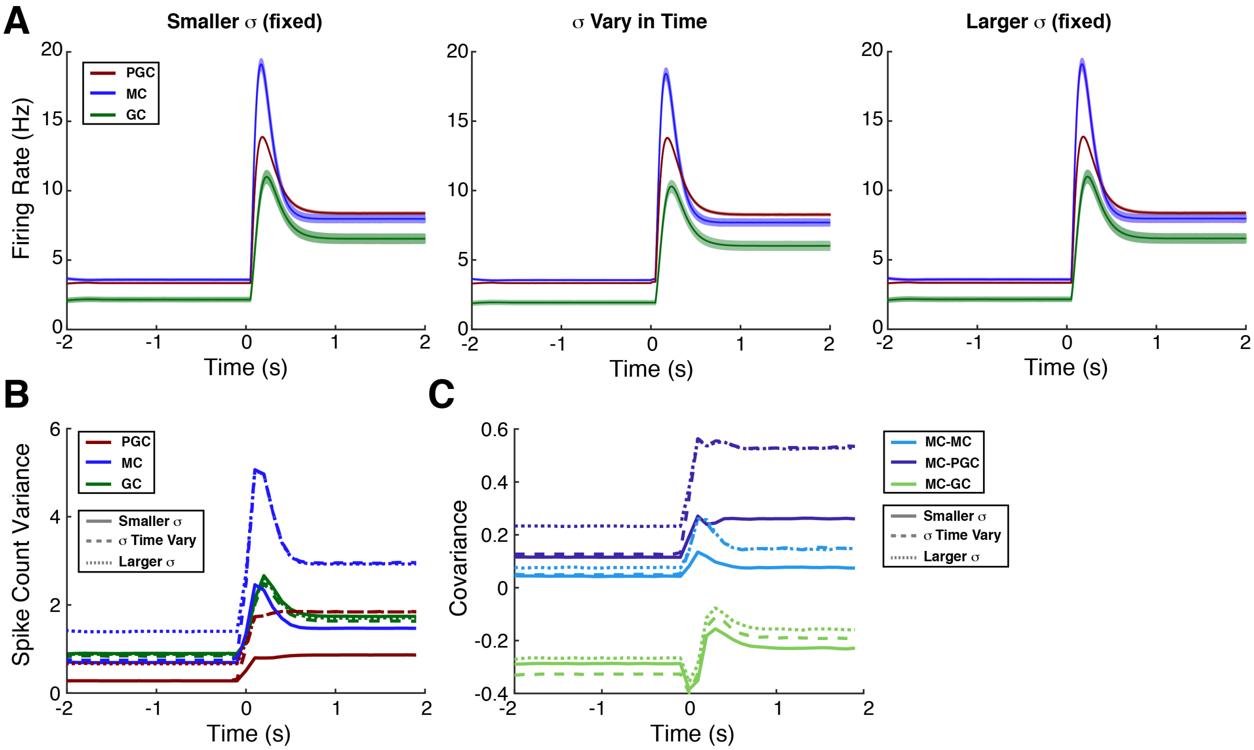

Figure S2: **Spike statistics of other cell types in model, Related to Figure 3.** Firing rate model results for 3 input noise regimes, averaging over the 10 best sets of coupling strengths (Fig. 3**D** in main text). These model results are consistent with experiments, see **Input statistics give reasonable model results**. **A)** Firing rate for 3 different cell types in the firing rate model, shaded error bars representing standard deviation across 10 best parameter sets. **B)** Spike count variance for 3 different cell types, showing 3 different input noise regimes. **C)** Spike count covariance for 3 pairs of cell types, again combining 3 input noise regimes. Standard deviations (shaded regions) are omitted in **B)** and **C)** because they are small.

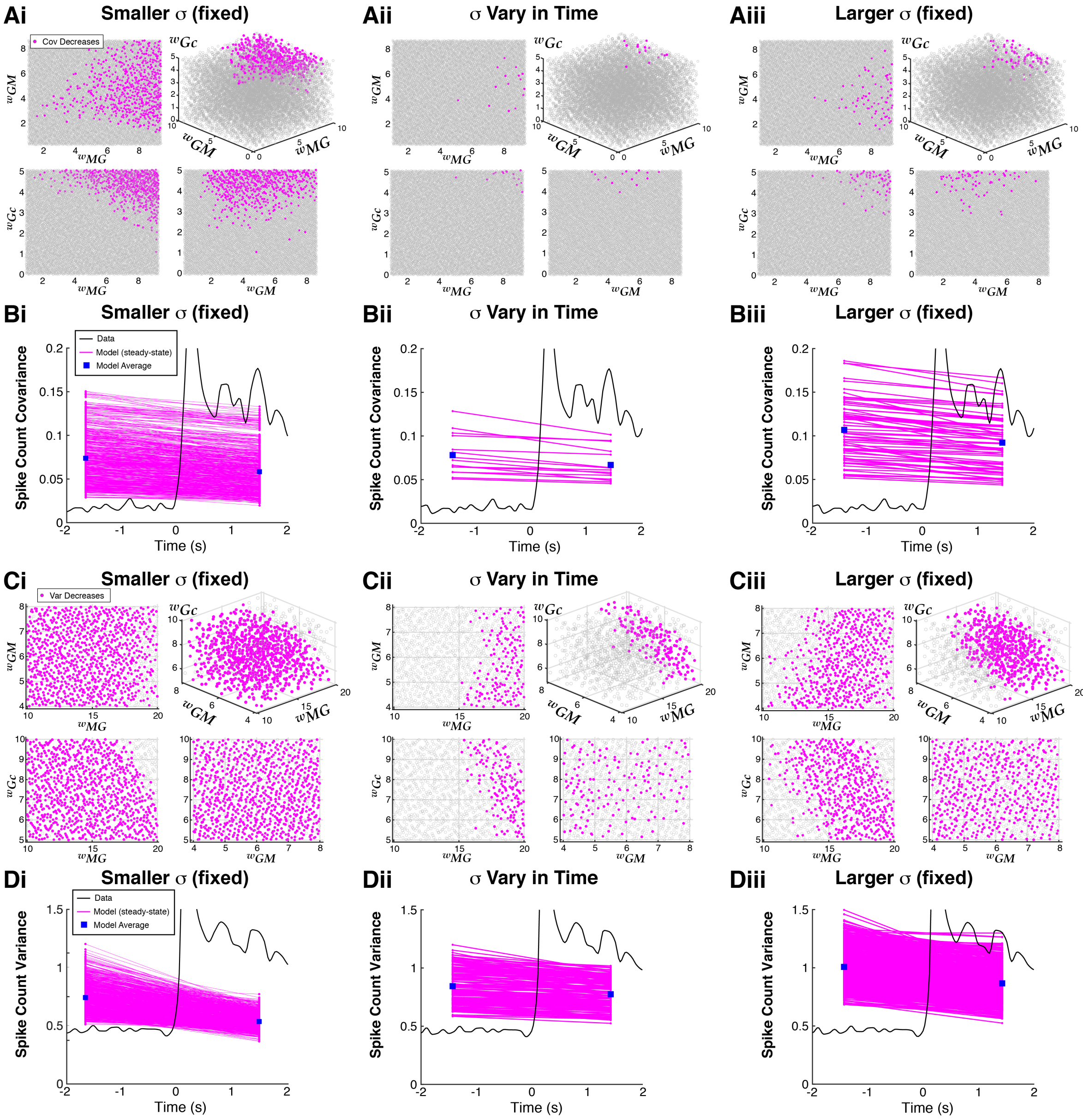

Figure S3: **Figure related to sub-section "Firing rate model can exhibit odor-evoked decreases in variability". A)** Parameters where covariance decreases with stimulus and mean spike count increases (magenta dots) from the entire set of 10,000 points and for all 3 input noise regimes. Results vary depending on input noise regime, but both the independent and common GC inhibition must be relatively strong while there does not appear to be a dependence on MC excitation. **B)** Covariance values when evoked is less than spontaneous (magenta), plotted with data (black); the blue squares denote averages of the model results. **C)** With much larger GC inhibition than before, stimulus-induced decreases in variance and increases in mean spiking (magenta dots) can occur. For **C)** and **D)** we test 1000 points with the inhibition values twice as large: $10 \leq w_{MG} \leq 20$, $5 \leq w_{Gc} \leq 10$, and a subset of excitation values $4 \leq w_{GM} \leq 8$. **Ci**) Small fixed σ easily results in evoked decreases in variance. For time-varying σ (**Cii**) and large fixed σ (**Ciii**), the inhibition has to be relatively large for evoked decreases in variance. In all of the (w_{MG}, w_{Gc}) plots, the upper right corner is gray because when total GC inhibition is excessive, the mean spike counts decrease in the evoked state. **D)** All variance values plotted with data (same format as **B**)).

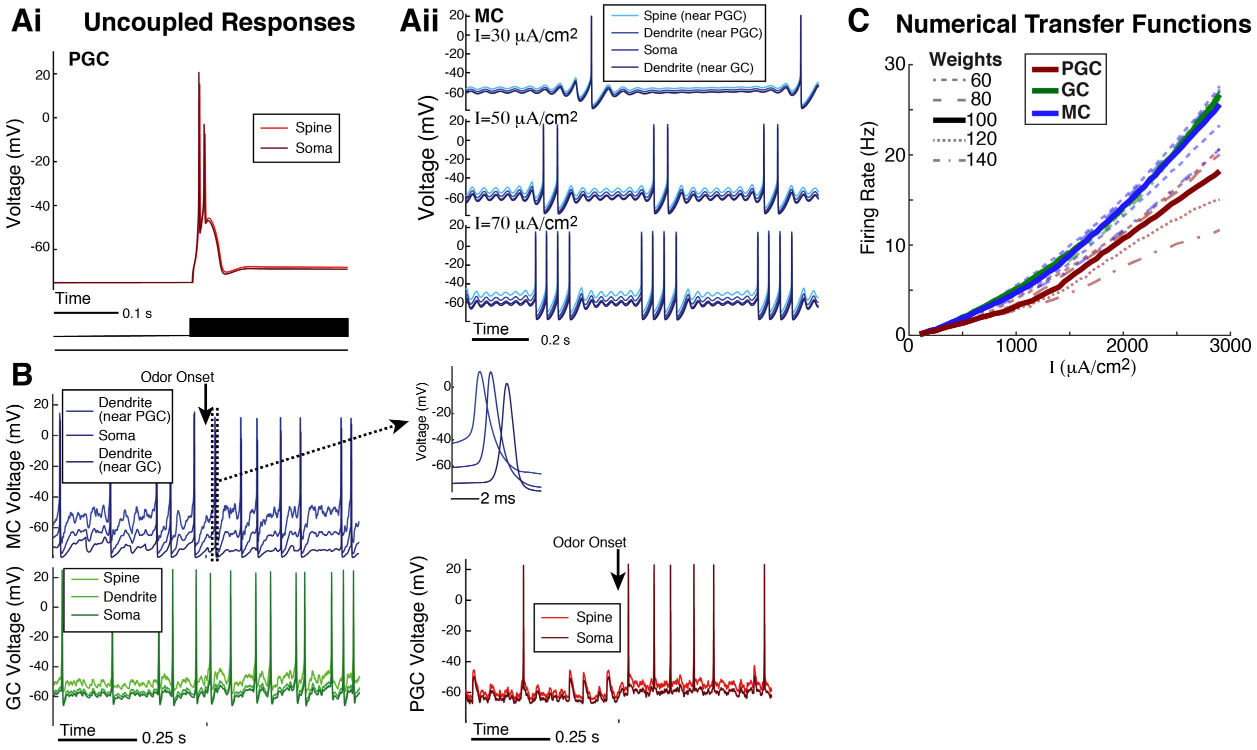

Figure S4: **Multi-compartment biophysical OB model used for the firing rate model, Related to firing rate model used in Figures 2, 3, 4, 6.** Small network of Cleland OB models (Li and Cleland, 2013, 2017). **A**) The firing rate response of uncoupled cells capture some known behavior observed in experiments. i) From rest (no current applied), an abrupt step of current injection to 50 μA/cm^2 results in transient spikes but no sustained firing, consistent with Li and Cleland (2013); McQuiston and Katz (2001). ii) Uncoupled MC show prominent sub-threshold oscillations (Desmaisons et al., 1999; Balu et al., 2004) and an increase in number of spikes per burst as applied current values increase, consistent with experiments in (Balu et al., 2004). **B**) Voltage traces of the coupled network show random spontaneous and evoked firing; inset of MC at bottom shows relatively fast propagation within a cell, on the order of a few milliseconds (Xiong and Chen, 2002). The results in **A**) and **B**) are not surprising because Li and Cleland (2013, 2017) showed these. **C**) Numerically computed transfer functions with several coupling strengths. Since the shape of the curves are qualitatively similar, we set the curves to be the solid thick curves ($w = 100$) in the firing rate model.
